# Association between the *EHBP1* SNPs and dyslipidemia in the end-stage renal disease patients with dialysis in Chinese Han population

**DOI:** 10.1186/s12944-024-02407-3

**Published:** 2024-12-27

**Authors:** Yan-Fei Lai, Zhong-E Liang, Chun-Xiang Wu, Min Zhang, Zong-Hu Shi, Xiao-Yan Meng, Chun-Xiao Liu

**Affiliations:** 1 Department of Nephrology, The Fourth Affiliated Hospital, Guangxi Medical University, Liuzhou, Guangxi 545005 People’s Republic of China; 2 Department of Prevention and Health Care, The Fourth Affiliated Hospital, Guangxi Medical University, Liuzhou, Guangxi 545005 People’s Republic of China

**Keywords:** *EHBP1*, Single nucleotide polymorphism, Dyslipidemia, End-stage renal disease

## Abstract

**Background:**

Lipid metabolism is influenced by mutations in the EH domain binding protein 1 gene (*EHBP1*). This study investigated the link between the *EHBP1* single-nucleotide polymorphisms (SNPs) and dyslipidemia risks in maintenance dialysis patients with end-stage renal disease in Chinese Han population.

**Methods:**

A total of 539 patients were divided into dyslipidemia (379) and control (160) groups. The patients with dyslipidemia were divided into four subgroups: high low-density lipoprotein cholesterol, low high-density lipoprotein cholesterol (HDLC), high triglyceride (TG) and high total cholesterol groups. The genotype distributions of three *EHBP1* SNPs (rs2710642, rs10496099 and rs1168816) were determined by high-throughput sequencing technology and were analyzed via generalized multifactor dimension reduction and binary logistic regression analysis.

**Results:**

The high-TG and control groups differed in terms of the genotype frequency of the rs2710642. One haplotype was detected in both the dyslipidemia and high-TG groups. The risk of dyslipidemia was 2.72-fold higher in participants with rs2710642GG compared with those of rs2710642AA and 2.62-fold higher compared with those with rs2710642AA + GA. Subjects who carried rs2710642GG had a 2.94 times greater risk of high TG levels than those who carried rs2710642AA and a 2.89 times greater risk than those who carried rs2710642AA + GA. Compared with those who carried rs2710642AA + GA, those who carried rs2710642GG were 2.53 times more likely to have low HDLC levels. The rs2710642–body mass index (BMI) (≥ 24 kg/m^2^) and rs11688816A–rs2710642G haplotype interactions increased the risk of dyslipidemia, and the rs2710642–BMI (≥ 24 kg/m^2^) interaction increased the risk of high TG levels. The rs10496099–rs2710642 and rs10496099–rs2710642–rs11688816 interactions increased the risk of low HDLC levels.

**Conclusions:**

These results suggest that the *EHBP1* rs2710642G and rs2710642GG and interactions with rs11688816A or BMI (≥ 24 kg/m^2^) were linked to higher dyslipidemia risks in end-stage renal disease patients in Chinese Han population.

**Supplementary Information:**

The online version contains supplementary material available at 10.1186/s12944-024-02407-3.

## Background

Chronic kidney disease consists of different stages, with the fifth one being end-stage renal disease (ESRD) characterized by a glomerular filtration rate of less than 15 mL/min∙1.73 m^2^ or on dialysis. Patients with ESRD and cardiovascular disease have greater mortality than those with cardiovascular disease alone or other patients with normal renal function [1]. In 2016, patients undergoing regular dialysis treatment had an overall mortality of 18.4%, of whom 43% passed away as a result of cardiovascular conditions [2]. The hyperlipidemia indicates that an individual has abnormally elevated levels of serum lipids or lipoproteins, leading to abnormal fat metabolism or function; notably, various lipids, such as high- and low-density lipoproteins, total cholesterol (TC) as well as TG regulate microvascular functions [3, 4]. Dyslipidemia is significantly correlated with cardiovascular disease [5, 6]. Moreover, dyslipidemia is a common complication of chronic kidney disease [7–10]. In ESRD patients, alterations in the internal environment can induce changes in the quantity and quality of circulating lipoproteins. Studies have shown that dyslipidemia in chronic kidney disease patients is characterized by low HDLC levels and high TG levels, as well as other compositional changes in lipoproteins [11–14]. In ESRD patients, lipid metabolism is disrupted, and increased serum TG, TC, high low-density lipoprotein cholesterol (LDLC) levels increase the risk of atherosclerosis [15, 16]. HDLC is involved in the cholesterol reverse transport process to remove excess cholesterol particles from peripheral tissues and is known to have a protective effect on the atherogenic process [17, 18]. The synthesis and maturation of high-density lipoprotein in ESRD patients are disrupted in several ways [7, 19]. Obesity, smoking, diabetes and a high-fat diet have been traditionally linked to a higher risk of dyslipidemia, and thus have received widespread attention and warrant early intervention. Nontraditional risk factors leading to dyslipidemia, such as internal environmental factors in ESRD patients and blood lipid-related genetic variation, have rarely been studied.

The epidermal growth factor receptor pathway substrate 15 homeodomain-binding protein, highly expressed in adipose tissue, is encoded by the *EHBP1* gene, and it has multiple transcript variants as a result of alternative splicing [20]. *EHBP1* plays a central role in fat cell transport [21]. In addition, *EHBP1* is crucial for the autophagic digestion of lipid droplets during lipophagy in hepatocytes [22]. A mutation in the rs2710642 locus was found to be associated with changes in low-density lipoprotein levels in a genome-wide association study involving 180,000 European individuals [23]. One study suggested that the *EHBP1* rs2710642 was linked to the odds of having dyslipidemia in ischemic stroke patients [24]. The previous study revealed that the *EHBP1* rs2710642A allele and the rs10496099C allele may be beneficial for normal levels of lipids in the Han Chinese population, the rs2710642G–rs10496099C haplotype was associated with a 2.64-fold increase in dyslipidemia risks, while the rs2710642G–rs10496099C haplotype together with hypertension and elevated fasting glucose (7.0 mmol/L or higher) raised the risk of dyslipidemia by 2.90- and 2.97-fold, respectively [25]. The human body mass index locus of the gene may be associated with rs11688816, which has been shown to be a significant phenotype in the fly [26]. However, it remains unclear how *EHBP1* SNPs are linked to hyperlipidemia risks in ESRD patients. The expression of the same gene is affected by different environmental factors, and its expression may vary significantly across different populations and diseases [27, 28]. Therefore, the current research explored how *EHBP1* SNPs (rs2710642, rs2710642 and rs11688816), SNP–SNP interactions, and gene‒environment interactions were associated with dyslipidemia risks in ESRD patients in the Chinese Han population.

## Methods

### Participants

A total of 539 ESRD patients in Chinese Han population were randomly recruited between January 2021 and December 2022 from the Fourth Affiliated Hospital of Guangxi Medical University, including 160 normolipidemic patients (control) and 379 dyslipidemia patients, the latter of which were divided into four subgroups: high-TC, high-TG, high-LDLC and low-HDLC groups. The participants were aged between 18 and 80 years, with each group having equivalent mean ages and gender ratios (*P* > 0.05). Specifically, the control group had a mean age of 58.15 ± 12.91 years, with 87 (54.37%) and 73 (45.63%) subjects being males and females, respectively, while for the dyslipidemia group, the mean age was 57.18 ± 12.63 years, of which 221 subjects (58.31%) were males and 158 subjects (41.69%) were females; for the 63 subjects of the high-TC group, the mean age was 56.37 ± 13.57 years, and 26 subjects (41.27%) were males and 37 subjects (58.73%) were females; for the 229 subjects in the high-TG group, the mean age was 56.81 ± 11.60 years, and 124 subjects (54.15%) were males and 105 subjects (45.85%) were females; for the 57 subjects in the high-LDLC group, the mean age was 54.98 ± 14.51 years, and 25 subjects (43.86%) were males and 32 subjects (56.14%) were females; and for the 309 subjects in the low-HDLC group, the mean age was 57.50 ± 12.53 years, and 191 subjects (61.81%) were males and 118 subjects (38.19%) were females. Hypertension was assessed via three or more blood pressure measurements using standard methods before hemodialysis on the day of dialysis, when the patient was quiet, awake and not on antihypertensive drugs. For all participants, blood samples (3 mL) were collected following an 8-h fasting period to detect the serum levels of lipids and fasting blood glucose.

The criteria for recruitment were as follows: (a) ESRD was diagnosed when the eGFR was < 15 mL/(min·1.73 m^2^); (b) maintenance of regular dialysis was consistent for more than 3 months; (c) no medication was taken to regulate blood lipids; (d) no other conditions/diagnoses, such as pregnancy, malignancy, active infection, autoimmune disease or liver disease were present; and (e) acute kidney injury and nephrotic syndrome were not present. All participants provided informed consent for this study which was approved by the Ethics Committee of the Fourth Affiliated Hospital of Guangxi Medical University (No. KY2021002).

## Detection of serum lipid levels

Dyslipidemia was determined according to the Guidelines for the Management of Dyslipidemia [29, 30] and the normal range standard of the Laboratory Department of the Fourth Affiliated Hospital of Guangxi Medical University (ISO15189 certification). An autoanalyzer (type Cobas 8000; Roche, Shanghai, China) was used for measurements, with commercial enzymatic assay kits used to determine the serum levels of LDLC, HDLC, TG and TC. The reference ranges for the serum levels of LDLC, HDLC, TG and TC were < 3.37 mmol/L, > 1.04 mmol/L, < 1.70 mmol/L and < 5.18 mmol/L, respectively.

### Genotyping

The gene and SNPs were selected and analyzed as follows. ① A GWAS dataset, related to lipid metabolism, was extracted for the *EHBP1* gene. ②Haploview (Broad Institute of MIT and Harvard, Cambridge, MA, United States version 4.2) was then used to select SNPs linked to lipid metabolism. ③ The NCBI dbSNP Build 132 (http://www.ncbi.nlm.nih.gov/snp/) was used to get information for each SNP. ④ Those having allele frequencies ≥ 0.05 were included. ⑤ The *EHBP1* SNPs (rs2710642, rs10496099 and rs1168816) that could be linked to blood lipid levels, as previously reported, were included.

Blood samples (539), obtained from the Fourth Affiliated Hospital of Guangxi Medical University, were used for isolating genomic DNA via the phenol‒chloroform method. Genotyping was then performed on a 10-μL DNA sample (10 ng/μL) from each participant at the Next-Generation Sequencing Department of Biotechnology Ltd. (Shanghai, China). For the three SNPs, the following sense and antisense primers were used: rs10496099F 5′-TGGAATCACATCTGGACA-AGATTTTGC-3′, rs10496099R 5′-CATTTCTCTCCTTGGCTTCTATGACTC-3′, rs11688816F 5′-CATATTGATGCTGCTAGTAGCAAGA-3′, rs11688816R 5′-CTGCCTGGGTTACCGCTTTCCAATT-3′, rs2710642F 5′-TCTTTGTCCTTTTC-ATCTTTATGTTGAGTA-3′, and rs2710642R 5′-GTCTTTTCACTTTCAACGTATTTGTGTCTT-3′.

## Statistical analysis

All results were statistically analyzed using SPSS 27.0 software, with normally distributed data presented as mean ± standard deviation before analysis using *t-*test for two-group comparisons. The variables with a skewed distribution are expressed as medians and quartiles, and two-group comparisons were performed with the Mann‒Whitney nonparametric test. Differences in count data were analyzed using chi-square test. Haplotype analysis was conducted, and the link between genotype and the risk of dyslipidemia were tested via SNPStats online software. The SHEsis online software (http://analysis.bio-x.cn/myAnalysis.php) was used to calculate the Hardy‒Weinberg equilibrium (HWE), linkage disequilibrium of the *EHBP1* SNPs, denoted as *D*′ and *R* ^2^ values. Optimal SNP‒SNP as well as haplotype‒haplotype interaction models between *EHBP1* loci were explored with the generalized multifactor dimension reduction software v0.7 before screening SNP‒environment interaction models. Odds ratios (OR) and 95% confidence intervals (CI) were then calculated with binary logistic regression. All other data graphics were generated via GraphPad Prism (version 8.0.0). Statistical significance was considered at *P* < 0.05.

## Results

### General and biochemical features of the participants

As shown in sTable 1 (Additional file 1), gender, age, height, systolic blood pressure or the proportion of patients with hypertension were not different between the control and dyslipidemia subgroups (*P* > 0.05), while the dyslipidemia, low-HDLC and high-TG groups had significantly greater average weight, BMI and fasting blood sugar (FBS) level compared with the control (*P* < 0.05). The proportion of patients with diabetes was significantly greater in the low-HDLC and high-TG groups compared with the control group (*P* < 0.05).

### Associations between the *EHBP1* genotypes and the risk of dyslipidemia in ESRD patients

The minor allele frequencys of three SNPs (rs10496099, rs11688816 and rs2710642) in the control and case groups were greater than 0.05 (Table 1), with their allelic and genotypic distributions maintaining concordance with the HWE in the control and all dyslipidemia groups (*P* > 0.05 for all). The rs2710642 of the high-TG and control groups had significantly different genotypic distributions (*P* = 0.038), and the frequencies of rs2710642G (32% *vs*. 26%) and rs2710642GG (12% *vs*. 4%) in the high-TG group were greater compared with the control group. However, the genotypic distributions of the rs10496099 and rs11688816 did not differ between the dyslipidemia and control group (*P* > 0.05).

**Table 1:** Genotypic and allelic frequencies of the *EHBP1* SNPs in the control and disease groups [n (%)]

SNP/ Genotype/ Allele	Control group (*n* = 160)	Dyslipidemia group (*n* = 379)	TC^a^ (*n* = 63)	LDLC^b^ (*n* = 57)	TG^c^ (*n* = 229)	HDLC^d^ (*n* = 309)	*P*_D_ _*value*_	*P *_TC_ _*value*_	*P*_LDLC_ _*value*_	*P*_TG_ _*value*_	*P*_HDLC_ _*value*_
rs10496099											
CC	82 (51)	183 (48)	36 (57)	28 (49)	111 (49)	155 (50)					
CT	70 (44)	160(42)	20 (32)	22 (39)	95 (41)	127 (41)					
TT	8 (5)	36 (10)	7 (11)	7 (12)	23 (10)	27 (9)	0.217	0.201	0.298	0.195	0.337
C	234 (73)	526 (69)	92 (73)	78 (68)	317 (69)	437 (71)					
T	86 (27)	232 (31)	34 (27)	36 (32)	141 (31)	181 (29)	0.220	0.981	0.337	0.238	0.437
* P*_HWE_	0.153	0.905	0.123	0.419	0.688	0.892					
rs11688816											
AA	20 (12)	60 (16)	9 (14)	7 (13)	39 (17)	45 (14)					
GA	62 (39)	161 (42)	21 (33)	23 (40)	97 (42)	126 (41)					
GG	78 (49)	158 (42)	33 (53)	27 (47)	93 (41)	138 (45)	0.286	0.746	0.977	0.224	0.665
A	102 (32)	281 (37)	39 (31)	37 (32)	177 (38)	216 (35)					
G	218 (68)	477 (63)	87 (69)	77 (68)	281 (62)	402 (65)	0.103	0.850	0.909	0.053	0.345
* P*_HWE_	0.173	0.081	0.081	0.547	0.119	0.069					
rs2710642											
AA	85 (53)	183 (48)	37 (59)	30 (53)	110 (48)	157 (51)					
GA	68 (43)	156 (40)	19 (30)	20 (35)	92 (40)	121 (39)					
GG	7 (4)	40 (11)	7 (11)	7 (12)	27 (12)	31 (10)	0.064	0.141	0.188	0.038	0.102
A	238 (74)	522 (69)	93 (74)	80 (70)	312 (68)	435 (70)					
G	82 (26)	236 (31)	33 (26)	34 (30)	146 (32)	183 (30)	0.070	0.902	0.384	0.059	0.199
* P*_HWE_	0.146	0.435	0.08	0.222	0.256	0.286					

^a^TC, high TC group. ^b^LDLC, high LDLC group. ^c^TG, high TG group. ^d^HDLC, low HDLC group. HWE means Hardy−Weinberg equilibrium. *P*_*D*_ *-value,* The *P-value* for the dyslipidemia group. *P* *-value* < 0.05 indicated statistically significant difference

### Associations between the genetic models of *EHBP1* genotypes and the risk of dyslipidemia in ESRD patients

Genetic models were constructed to detect the associations between the three SNP genotypes and dyslipidemia risks in ESRD patients (Table 2). The rs2710642GG genotype was significantly and positively associated with dyslipidemia risks. In the codominant and recessive models, the risk of dyslipidemia in subjects carrying rs2710642GG was 2.72 times greater than that in subjects carrying rs2710642AA (95% CI = 1.17–6.35, *P*= 0.04), and the risk was 2.62 times greater in the subjects who carried rs2710642GG than in those who carried rs2710642AA+GA (95% CI = 1.15–6.00,*P* = 0.012). Furthermore, as shown in Table 3, similar positive associations were observed among the high-TG, low-HDLC and control groups. The risk of high TG levels in subjects who carried rs2710642GG was 2.94 times greater than that in those who carried rs2710642AA (95% CI = 1.21–7.11, *P*= 0.033) and 2.89 times greater than that in those who carried rs2710642AA+GA (95% CI = 1.22–6.83,*P* = 0.009). Moreover, the risk of low HDLC in subjects who carried rs2710642GG was 2.53 times greater than that in those who carried rs2710642AA+GA (95% CI = 1.08–5.89,*P* = 0.02).

**Table 2:** Association between the *EHBP1* genotypes and the risk of dyslipidemia in ESRD patients [n (%)]

Loci	Model	Genotype	Control group (*n*=160)	Dyslipidemia group (*n*=379)	OR (95% CI)	*P-value*
rs10496099	Codominant	C/C T/C T/T	82 (51.2) 70 (43.8) 8 (5)	183 (48.3) 160 (42.2) 36 (9.5)	1.00 1.04 (0.71-1.53) 2.04 (0.90-4.59)	0.19
	Dominant	C/C T/C-T/T	82 (51.2) 78 (48.8)	183 (48.3) 196 (51.7)	1.00 1.04 (0.71-1.66)	0.49
	Recessive	C/C-T/C T/T	152 (95) 8 (5)	343 (90.5) 36 (9.5)	1.00 2.00(0.91-4.42)	0.068
	Overdominant	C/C-T/T T/C	90 (56.2) 70 (43.8)	219 (57.8) 160 (42.2)	1.00 0.95 (0.65-1.38)	0.78
rs11688816	Codominant	G/G G/A A/A	78 (48.8) 62 (38.8) 20 (12.5)	158 (41.7) 161 (42.5) 60 (15.8)	1.00 1.29 (0.86-1.93) 1.47 (0.82-2.61)	0.29
	Dominant	G/G G/A-A/A	78 (48.8) 82 (51.2)	158 (41.7) 221 (58.3)	1.00 1.33 (0.92-1.94)	0.13
	Recessive	G/G-G/A A/A	140 (87.5) 20 (12.5)	319 (84.2) 60 (15.8)	1.00 1.30 (0.75-2.24)	0.35
	Overdominant	G/G-A/A G/A	98 (61.2) 62 (38.8)	218 (57.5) 161 (42.5)	1.00 1.18 (0.81-1.72)	0.4
rs2710642	Codominant	A/A G/A G/G	85 (53.1) 68 (42.5) 7 (4.4)	183 (48.3) 156 (41.2) 40 (10.6)	1.00 1.08 (0.73-1.59) 2.72 (1.17-6.35)	0.04
	Dominant	A/A G/A-G/G	85 (53.1) 75 (46.9)	183 (48.3) 196 (51.7)	1.00 1.23 (0.85-1.79)	0.27
	Recessive	A/A-G/A G/G	153 (95.6) 7 (4.4)	339 (89.5) 40 (10.6)	1.00 2.62 (1.15-6.00)	0.012
	Overdominant	A/A-G/G G/A	92 (57.5) 68 (42.5)	223 (58.8) 156 (41.2)	1.00 0.95 (0.65-1.39)	0.8

The *P-value* was adjusted by gender and age, *P-value* < 0.05 indicated statistically significant difference

*CI *Confidence interval, *OR *Odds ratio

**Table 3:** Association between the rs2710642 genotypes and dyslipidemia subgroups in ESRD patients [n (%)]

Group	Loci	Model	Genotype	Control group	Case group	OR (95% CI)	*P-value*
Control *vs* HTC^a^	rs2710642	Codominant	A/A G/A G/G	85 (53.1) 68 (42.5) 7 (4.4)	37 (58.7) 19 (30.2) 7 (11.1)	1.00 0.58 (0.30-1.12) 1.92 (0.61-6.02)	0.079
		Dominant	A/A G/A-G/G	85 (53.1) 75 (46.9)	37 (58.7) 62 (41.3)	1.00 0.71 (0.39-1.30)	0.27
		Recessive	A/A-G/A G/G	153 (95.6) 7 (4.4)	56 (88.9) 7 (11.1)	1.00 2.43 (0.80-7.35)	0.12
		Overdominant	A/A-G/G G/A	92 (57.5) 68 (42.5)	44 (69.8) 19 (30.2)	1.00 0.54 (0.29-1.01)	0.05
Control *vs* HLDLC^b^	rs2710642	Codominant	A/A G/A G/G	85 (53.1) 68 (42.5) 7 (4.4)	30 (52.6) 20 (35.1) 7 (12.3)	1.00 0.75 (0.38-1.46) 2.45 (0.77-7.81)	0.14
		Dominant	A/A G/A-G/G	85 (53.1) 75 (46.9)	30 (52.6) 276 (47.4)	1.00 0.90 (0.48-1.69)	0.75
		Recessive	A/A-G/A G/G	153 (95.6) 7 (4.4)	50 (87.7) 7 (12.3)	1.00 2.82 (0.92-8.59)	0.073
		Overdominant	A/A-G/G G/A	92 (57.5) 68 (42.5)	37 (64.7) 20 (35.1)	1.00 0.66 (0.34-1.25)	0.2
Control *vs* HTG^c^	rs2710642	Codominant	A/A G/A G/G	85 (53.1) 68 (42.5) 7 (4.4)	110 (48) 92 (40.2) 27 (11.8)	1.00 1.04 (0.68-1.59) 2.94 (1.21-7.11)	0.033
		Dominant	A/A G/A-G/G	85 (53.1) 75 (46.9)	110 (48) 119 (52)	1.00 1.21 (0.80-1.82)	0.36
		Recessive	A/A-G/A G/G	153 (95.6) 7 (4.4)	202 (88.2) 27 (11.8)	1.00 2.89 (1.22-6.83)	0.009
		Overdominant	A/A-G/G G/A	92 (57.5) 68 (42.5)	137 (59.8) 92 (40.2)	1.00 0.90 (0.59-1.35)	0.6
Control *vs*LHDLC^d^	rs2710642	Codominant	A/A G/A G/G	85 (53.1) 68 (42.5) 7 (4.4)	157 (50.8) 121 (39.2) 31 (10)	1.00 0.99 (0.66-1.47) 2.51 (1.06-5.97)	0.068
		Dominant	A/A G/A-G/G	85 (53.1) 75 (46.9)	157 (50.8) 152 (49.2)	1.00 1.13 (0.77-1.66)	0.54
		Recessive	A/A-G/A G/G	153 (95.6) 7 (4.4)	278 (90) 31 (10)	1.00 2.53 (1.08-5.89)	0.02
		Overdominant	A/A-G/G G/A	92 (57.5) 68 (42.5)	188 (60.8) 121 (39.2)	1.00 0.89 (0.60-1.31)	0.54

The *P* *-value *was adjusted by gender and age, *P* *-value* < 0.05 indicated statistically significant difference

^a^HTC, high TC group. ^b^HLDLC, high LDLC group. ^c^HTG, high TG group. ^d^LHDLC, low HDLC group. CI, confidence interval. OR, odds ratio. The *P* *-value *was adjusted by gender and age, *P-value* < 0.05 indicated statistically significant difference

### Linkage disequilibrium and haplotype analysis

**Fig. 1:**
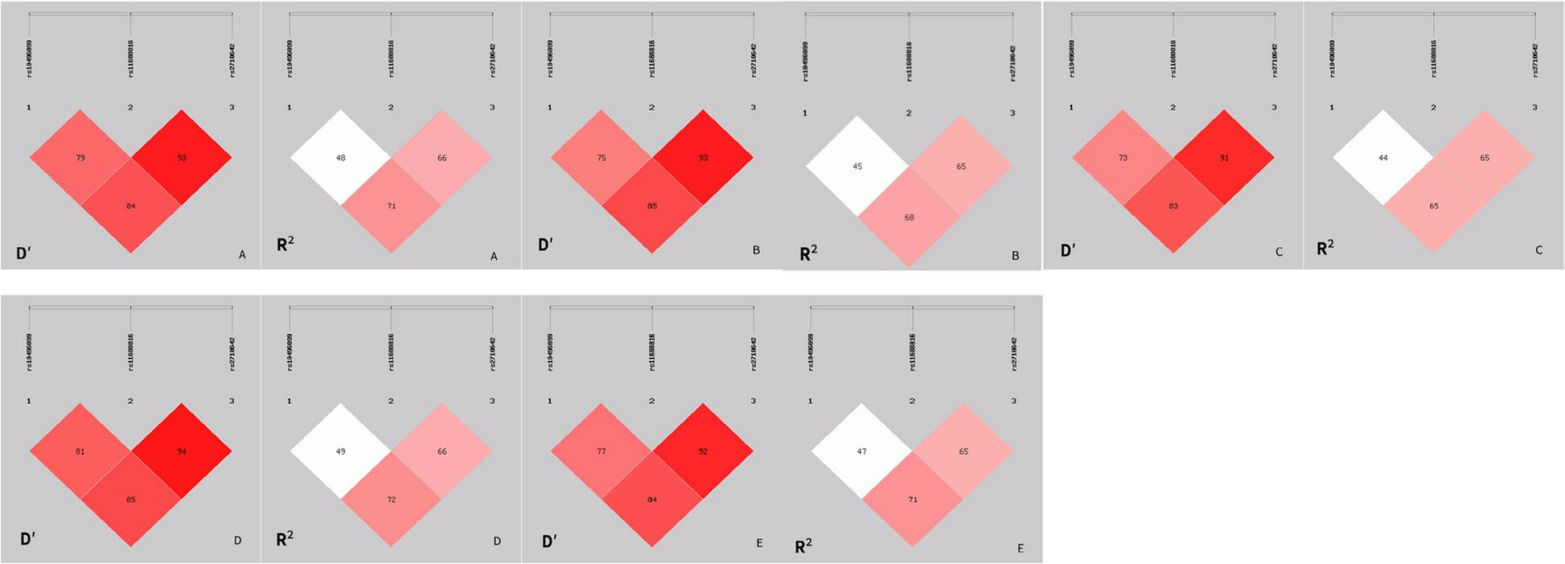
Linkage disequilibrium analysis for the three *EHBP1* SNPs in normal and dyslipidemia groups. A = dyslipidemia group. B = high TC group. C = high LDLC group. D = high TG group. E = low HDLC group

The three SNPs exhibited linkage disequilibrium in the control and dyslipidemia, high-TC, high-TG, high-LDLC and low-HDLC groups (Fig. 1). Strong linkage disequilibrium was noted between rs2710642 and rs11688816 as well as between rs2710642 and rs10496099 in the control and case groups (*D*′ ≥ 0.83 and* R* ^2^ ≥ 0.65). As shown in Table 4, the frequency of the rs2710642G–rs11688816A haplotype differed between the control and dyslipidemia groups. The subjects who carried rs2710642G–rs11688816A had a 1.38 times greater dyslipidemia risk (95% CI = 1.01–1.90, *P* = 0.047) and a 1.41 times greater TG risk (95% CI = 1.00–1.99, *P* = 0.048) than those who carried rs2710642A–rs11688816G.

**Table 4:** Analysis of the *EHBP1* rs10496099, rs11688816 and rs2710642 SNPs haplotypes

Group	Haplotype	Freq	Control	Case	OR (95% CI)	*P* *-value*
Ctrl *vs* Dyslipidemia group	rs11688816G - rs2710642A	0.632	0.668	0.618	1.00	-
	rs11688816A - rs2710642G	0.283	0.243	0.300	1.38 (1.01 - 1.90)	0.047
Ctrl *vs* high TG group	rs11688816G - rs2710642A	0.633	0.668	0.609	1.00	-
	rs11688816A - rs2710642G	0.282	0.243	0.309	1.41 (1.00 - 1.99)	0.048

*P*-value < 0.05 indicated statistically significant difference

*Ctrl *Control, *CI *Confidence interval, *OR *Odds ratio

### Different interaction models for the risk of dyslipidemia

Several models of the effects of haplotype–haplotype, haplotype–environment, SNP–SNP and SNP–environment interactions on the risk of dyslipidemia were analyzed via generalized multifactor dimension reduction (Table 5). Six optimal models were significantly associated with the risk of dyslipidemia, and their CV constancy was 10/10, the balanced accuracy test was > 50%, and the sign test *P* value was < 0.05 for all models. SNP‒SNP interactions of rs10496099‒rs2710642 and rs10496099‒rs11688816‒rs2710642 increased low HDLC risk (*P* = 0.001). SNP‒environment interactions of rs2710642–BMI (≥ 24 kg/m^2^) increased dyslipidemia (*P* = 0.010), rs2710642–BMI and rs10496099–rs11688816–BMI (≥ 24 kg/m^2^)–gender (female)–age (≥ 65 years)–FBS (≥ 7.0 mmol/L) increased high TG risk (*P* = 0.001 and *P* = 0.011, respectively), and rs10496099–rs2710642–BMI‒gender increased low HDLC risk (*P* = 0.001).

**Table 5:** Different interaction models related to dyslipidemia in ESRD patients

Interactive model	Training bal. acc.	Testing bal. acc.	CV consistency	Sign test *P*
**Dyslipidemia group**				
SNP-SNP				
A-B	0.5496	0.5232	8/10	0.623
A-B-C	0.5655	0.5228	10/10	0.377
SNP-Environment				
C-D	0.5956	0.5857	10/10	0.010
A-C-D	0.6084	0.5625	7/10	0.054
**High TG group**				
SNP-SNP				
A-B-C	0.5732	0.5310	10/10	0.171
SNP-Environment				
C-D	0.6448	0.6447	10/10	0.001
A-B-D-E-F-G	0.7196	0.5570	10/10	0.011
B-C-D	0.6561	0.6334	9/10	0.001
Haplotype-Haplotype				
H1-H2	0.5327	0.4999	10/10	0.377
Haplotype-Environment				
H1-H2-D	0.6355	0.6126	7/10	0.001
H1-D-E-F-G	0.6590	0.5545	7/10	0.011
**High LDLC group**				
SNP-SNP				
A-B-C	0.5813	0.4392	10/10	0.945
SNP-Environment				
A-B-E	0.6347	0.4848	8/10	0.623
**High TC group**				
SNP-SNP				
A-B-C	0.5969	0.5220	10/10	0.623
SNP-Environment				
A-B-D-E-F-G	0.7423	0.5338	8/10	0.377
**Low HDLC group**				
SNP-SNP				
A-C	0.7656	0.7408	10/10	0.001
A-B-C	0.7760	0.7596	10/10	0.001
SNP-Environment				
A-C-D-E	0.8156	0.7901	10/10	0.001
A-C-D	0.7994	0.7787	9/10	0.001

A = rs10496099. B = rs11688816. C = rs2710642. D = BMI ≥ 24kg/m^2^. E = gender (Female). F = age ≥ 65years. G = FBS ≥ 7.0mmol/L. H1 = rs11688816A - rs2710642G. H2 = rs10496099T - rs116688816G. BMI, body mass index. Bal. Acc., balanced accuracy. CV, cross-validation. FBS, fasting blood sugar. *P* *-value* < 0.05 indicated statistically significant difference

Furthermore, subjects who carried rs2710642GG and had a BMI ≥ 24 kg/m^2^ had an 8.92-fold greater dyslipidemia risk than those who carried 2710642GA + AA and had a BMI < 24 kg/m^2^ (95% CI: 1.169–68.056, *P* = 0.035), and subjects who carried rs2710642GA + AA and had a BMI ≥ 24 kg/m^2^ had a 2.333 times greater dyslipidemia risk than those who carried rs2710642GA + AA but had a BMI < 24 kg/m^2^ (95% CI: 1.454–3.745, *P* < 0.001). The subjects who carried rs2710642GG and had a BMI ≥ 24 kg/m^2^ had a 14.368-fold greater high TG risk than those who carried 2710642GA + AA and had a BMI < 24 kg/m^2^ (95% CI: 1.850–111.575,* P* = 0.011), and subjects who carried rs2710642GA + AA and had a BMI ≥ 24 kg/m^2^ had a 3.602 times greater high TG risk than those who carried rs2710642GA + AA but had a BMI < 24 kg/m^2^ (95% CI: 2.185–5.940,* P* < 0.001). The subjects who carried rs2710642GG and rs10496099TT had a 3.347-fold greater risk of low HDLC than those who carried 2710642GA + AA and rs10496099TC + CC (95% CI: 1.153–2.915,* P* = 0.026; Table 6).

**Table 6:** Meaningful bivalent interactive models of dyslipidemia in the ESRD patients

Group	Variable 1	Variable 2	*P* *-value*	OR (95% CI)
Ctrl *vs*Dyslipidemia	rs2710642	BMI ≥ 24kg/m^2^		
	GA+AA	no	-	1
	GG	yes	0.035	8.920 (1.169-68.056)
	GA+AA	Yes	<0.001	2.333 (1.454-3.745)
	GG	No	0.088	2.230 (0.888-5.600)
Ctrl *vs*HTG	rs2710642	BMI ≥ 24kg/m^2^		
	GA+AA	no	-	1
	GG	yes	0.011	14.368 (1.850-111.575)
	GA+AA	Yes	<0.001	3.602 (2.185-5.940)
	GG	No	0.061	2.579 (0.959-6.936)
Ctrl *vs*LHDLC	rs2710642	rs10496099		
	GA+AA	TC+CC	-	1
	GG	TT	0.026	3.374 (1.153-9.877)
	GA+AA	TT	0.266	0.360 (0.059-2.178)
	GG	TC+CC	0.747	0.810 (0.225-2.915)

*BMI *Body mass index, *Ctrl *Control, *CI *Confidence interval, *Dyslipidemia* Dyslipidemia group, *HTG *High TG group, *LHDLC *Low HDLC group, *OR *Odds ratio. *P-value* < 0.05 indicated statistically significant difference

### Risk factors for dyslipidemia in ESRD patients

**Fig. 2:**
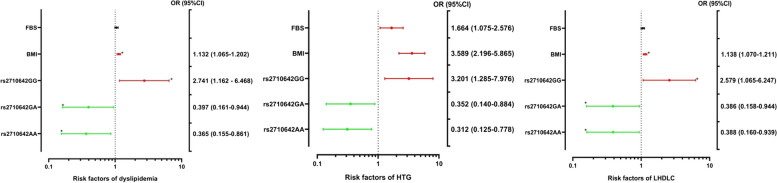
Associations of the stratified risk factors with different groups of dyslipidemia. Dyslipidemia, Dyslipidemia group. HTG, High TG group. LHDLC, Low HDLC group. BMI, body mass index. CI, confidence interval. FBS, fasting blood sugar. OR, odds ratio. The red colors represent risk factors, the green colors represent protective factors. *P-value < 0.05

As shown in Table 7 and Fig. 2, a BMI ≥ 24 kg/m^2^ increased the risk of dyslipidemia by 1.132 times (95% CI = 1.065–1.202, *P* < 0.001), with increased risks of low levels of HDLC as well as high levels of TG. FBS ≥ 7.0 mmol/L increased the risk of high TG levels by 1.664 times (95% CI = 1.075–2.576, *P* < 0.05). Subjects carried genotype rs2710642GG had a 2.741 times greater dyslipidemia risk than those who carried the rs2710642 AA genotype (95% CI = 1.162–6.468, *P* = 0.021), mainly indicated by an increased risk of low HDLC levels as well as high TG levels. In the high-TG and low-HDLC subgroups of the dyslipidemia group, the rs2710642GA or rs2710642AA genotype was a protective factor against dyslipidemia, low levels of HDLC and high levels of TG.

**Table 7:** Risk factors for dyslipidemia in ESRD patients

Group	Parameter	Control (n)	Case (n)	OR (95% CI)	*P-value*
Dyslipidemia	BMI	160	379	1.132 (1.065 - 1.202)	<0.001
	rs2710642^a^	160	379	0.365 (0.155-0.861)	0.021
	rs2710642 (1)	160	379	0.397 (0.161-0.944)	0.036
	rs2710642 (2)	160	379	2.741 (1.162 - 6.468)	0.021
HTG	BMI	160	229	3.589 (2.196-5.865)	<0.001
	rs2710642^a^	160	229	0.312 (0.125-0.778)	0.013
	rs2710642 (1)	160	229	0.352 (0.140-0.884)	0.026
	rs2710642 (2)	160	229	3.201 (1.285-7.976)	0.013
	FBS	160	229	1.664 (1.075-2.576)	0.022
LHDLC	BMI	160	309	1.138 (1.070-1.211)	<0.001
	rs2710642^a^	160	309	0.388 (0.160-0.939)	0.036
	rs2710642 (1)	160	309	0.386 (0.158-0.944)	0.037
	rs2710642 (2)	160	309	2.579 (1.065-6.247)	0.036

Dyslipidemia, Dyslipidemia group. HTG, High TG group. LHDLC, Low HDLC group. rs2710642^a^, AA. rs2710642 (1), GA. rs2710642 (2), GG. *BMI *Body mass index, *CI *Confidence interval, *FBS *Fasting blood glucose, *OR *Odds ratio. After univariate analysis, our variables included BMI, FBS, and rs2710642. *P-value* < 0.05 indicated statistically significant difference

## Discussion

A reduction in the glomerular filtration rate in ESRD patients changes the blood lipid profile, leading to dyslipidemia. Dyslipidemia is a sensitive risk factor for coronary heart disease or hypertension in susceptible individuals and is a common disease with complex pathogenesis. Dyslipidemia is associated with genetic factors, such as mutations in lipid-related genes, or interactions between different factors, including age, sex, diet, exercise and alcohol consumption [31–33], as well as gene‒environment interactions [34, 35]. According to Willer et al., the *EHBP1* rs2710642 exhibited a significant correlation with LDL-C levels in European individuals [23]. In this study, the rs2710642GG genotype was significantly positively associated with elevated serum TG levels and decreased HDLC levels in ESRD patients on dialysis in China. Ahmad S. et al. reported that the *EHBP1* rs11688816G allele was negatively correlated with BMI in 16,157 Pakistani individuals [36], and a larger-sample Mendelian randomization study revealed that the rs11688816G is an effect allele associated with increased BMI [37]. The Framingham Heart Study 100 K Project suggested that the rs10496099 is associated with the risk of atherosclerosis [38]. The previous studies also indicated a link between rs10496099T and high levels of TC and TG as well as low levels of HDLC in Chinese patients with ischemic stroke and coronary artery diseases [24]. In this study, the rs11688816 and rs10496099 were not independently associated with dyslipidemia risks in ESRD patients. It was previously reported that *EHBP1* SNP–SNP and SNP–environment interactions are involved in the pathogenesis of dyslipidemia. This study also found that the interaction of rs10496099 and rs11688816 with several environmental factors (BMI, gender, age and FBS) significantly increased the risk of high TG levels. Furthermore, the rs10496099–rs2710642 and rs10496099–rs2710642–rs11688816 interactions were linked to higher risks of low HDLC levels. The rs10496099–rs2710642–BMI (≥ 24 kg/m^2^)–female interaction increased the risk of low HDLC levels. These findings suggest that multiple SNP mutations or SNP‒environment interactions might lead to different dyslipidemia phenotypes in ESRD patients.

Previous research suggested that the effects of haplotypes on phenotypes were more pronounced compared with those induced by individual SNPs, with the link between haplotypes and phenotypes providing a better understanding of local ancestral genomic information and population genetic structure [24, 25, 39]. In this study, strong linkage disequilibrium was noted between the *EHBP1* rs11688816 and rs2710642 in the control and dyslipidemia groups, as well as between the *EHBP1* rs11688816 and rs2710642 in the control and high-TG groups. The haplotype rs11688816A–rs2710642G increased the risk of dyslipidemia by 1.38 times and increased the risk of high TG levels by 1.41 times. These results showed that the synergistic effect or haplotype of these SNPs is a better predictor than any single SNP in dyslipidemia risk models of ESRD patients.

Studies have shown that for overweight and even obese people, especially elderly people, in addition to the reasonable diet recommended by the American Heart Association, a weight cutoff of 5% to 10% can reduce the levels of LDL-C, TG, FBS and glycosylated hemoglobin and other risk factors for cardiovascular disease [40]. This study used logistic regression analysis to predict dyslipidemia risk. *EHBP1* mutation and BMI were found to be independent risk factors for increased dyslipidemia risk in ESRD patients. BMI increased dyslipidemia, high TG and low HDLC risk by 1.132, 3.589 and 1.138 times, respectively. Compared with rs2710642 AG genotype carriers, rs2710642GG genotype carriers had 2.741, 3.201 and 2.579 times greater dyslipidemia, high TG and low HDLC risk, respectively. Moreover, the synergistic interaction of rs2710642GG and BMI ≥ 24 kg/m^2^ significantly increased the risk of dyslipidemia by 8.920 times in ESRD patients, and BMI ≥ 24 kg/m^2^ was the dominant factor affecting the interaction. This result prompted us to investigate whether nurse-directed dietary adjustments for overweight ESRD patients who carried rs2710642GG reduced their risk of dyslipidemia and cardiovascular disease. This study found that the rs10496099 was not related to the risk of dyslipidemia in ESRD patients, whereas the synergistic interaction of genotypes rs2710642GG and rs10496099TT increased low HDLC risk by 3.374 times. Therefore, this study speculated that the haplotype of the two SNPs is a better predictor of low HDLC risk.

Proprotein convertase subtilisin/kexin type 9 gene (*PCSK9*) is crucial in cholesterol homeostasis by degrading hepatic low-density lipoprotein receptor, which leads to elevated serum lipid levels. The protein level of *EHBP1* was downregulated during *PCSK9* overexpression and upregulated during *PCSK9* knockdown in a mouse model [41]. Therefore, the study hypothesizes that mutations of lipid-related *EHBP1* SNPs may result in different lipid phenotypes and these SNPs might interact with the environment to alter dyslipidemia risk; moreover, they might interact with *PCSK9* to affect lipid levels. The study revealed that the rs2710642G allele, either independently or alongside FBS or BMI, was linked to dyslipidemia risks.

### Strengths and limitations

This study’s strength was the subgroup analysis combined with multiple models to comprehensively evaluate the effects of *EHBP1* SNPs (rs2710642, rs10496099, rs11688816), haplotypes, and environmental interactions on dyslipidemia in ESRD patients with dialysis at the molecular genetic level using high-throughput sequencing technology. However, this study was not without several limitations. Firstly, with this work being based on a single-center study, the results need to be verified with a larger sample size. Second, it did not investigate how *EHBP1* and dietary interactions influenced dyslipidemia risks in patients with ESRD. Third, the therapies for these ESRD patients included peritoneal dialysis and hemodialysis, but this study did not stratify the effects of the two methods on blood lipids, so a larger sample size is needed for further research.

## Conclusions

This study demonstrated that the *EHBP1* rs2710642G allele directly contributes to dyslipidemia and high TG levels in ESRD patients in Chinese Han population.. Haplotypes of *EHBP1* rs2710642 and rs11688816, interactions between three SNPs (rs10496099, rs2710642, and rs11688816) and environmental factors (BMI, FBS, etc.) altered the risk of dyslipidemia in ESRD patients. Therefore, *EHBP1* rs2710642G allele carried was a predict factor of dyslipidemia in ESRD patients in Chinese Han population, lose weight and BMI, and control their FBS might reduce their dyslipidemia risk.

## Supplementary Information

Supplementary Material 1.

## Data Availability

No datasets were generated or analysed during the current study.

## Abbreviations

BMI: Body mass index
CV: Cross-validation
CI: Confidence interval
ESRD: End-stage renal disease
*EHBP1*: EH domain binding protein 1 gene
FBS: Fasting blood sugar
HDLC: High density lipoprotein cholesterol
LDLC: Low density lipoprotein cholesterol
OR: Odds ratio
SNP: Single nucleotide polymorphism
*PCSK9*: Proprotein convertase subtilisin/kexin type 9 gene
TC: Total cholesterol
TG: Triglycerides
