# Supplementary material for: Association between the EHBP1 SNPs and dyslipidemia in the end-stage renal disease patients with dialysis in Chinese Han population
Source: Lipids Health Dis. 2024 Dec 27;23:422. doi: 10.1186/s12944-024-02407-3 (PMC11681726; doi:10.1186/s12944-024-02407-3)
Supplement: Supplementary file 1 — Supplementary Material 1. [file 12944_2024_2407_MOESM1_ESM.docx]

**sTable 1** General and biochemical characteristics of the participants.

| Parameter | Control  group | Dyslipidemia  group | TC^a^ | LDLC^b^ | TG^c^ | HDLC^d^ | *P*_D_  *value* | *P*_TC_  *value* | *P*_LDLC_  *value* | *P*_TG_  *value* | *P*_HDLC_  *value* |
| --- | --- | --- | --- | --- | --- | --- | --- | --- | --- | --- | --- |
| Number | 160 | 379 | 63 | 57 | 229 | 309 |  |  |  |  |  |
| Male/female | 87/73 | 221/158 | 26/37 | 25/32 | 124/105 | 191/118 | 0.399 | 0.078 | 0.173 | 0.965 | 0.120 |
| Age, years^e^ | 58.15 ± 12.91 | 57.18 ± 12.63 | 56.37 ± 13.57 | 54.98 ± 14.51 | 56.81 ± 11.60 | 57.50 ± 12.53 | 0.433 | 0.346 | 0.125 | 0.286 | 0.599 |
| Height, m | 1.61 ± 0.82 | 1.62 ± 0.07 | 1.59 ± 0.08 | 1.59 ± 0.09 | 1.61 ± 0.07 | 1.62 ± 0.07 | 0.268 | 0.525 | 0.245 | 0.794 | 0.117 |
| Weight, kg | 55.19 ± 9.28 | 59.17 ± 10.29 | 54.95 ± 8.95 | 55.90 ± 9.16 | 61.09 ± 10.59 | 59.97 ± 10.56 | <0.001 | 0.861 | 0.619 | <0.001 | <0.001 |
| BMI, kg/m2 | 21.27 ± 2.89 | 22.65 ± 3.50 | 21.60 ± 3.26 | 21.96 ± 2.92 | 23.51 ± 3.60 | 22.81 ± 3.62 | <0.001 | 0.464 | 0.125 | <0.001 | <0.001 |
| SBP, mmHg | 143.22 ± 21.16 | 140.39 ± 19.09 | 142.16 ± 21.71 | 144.30 ± 20.12 | 140.48 ± 19.06 | 140.60 ± 18.87 | 0.128 | 0.737 | 0.739 | 0.183 | 0.172 |
| DBP, mmHg | 80.45 ± 10.97 | 79.21 ± 9.92 | 78.44 ± 11.69 | 80.21 ± 10.92 | 79.23 ± 10.03 | 79.29 ± 9.65 | 0.199 | 0.229 | 0.887 | 0.002 | 0.242 |
| FBS, mmol/L^f^ | 2.82 (6.01) | 3.29 (6.66) | 4.47 (6.54) | 5.80 (2.81) | 3.73 (6.80) | 3.31 (6.68) | 0.013 | 0.206 | 0.993 | 0.001 | 0.009 |
| TC, mmol/L | 3.81 ± 0.69 | 4.12 ± 1.18 | 5.96 ± 0.77 | 5.71 ± 0.75 | 4.40 ± 1.04 | 3.86 ± 1.04 | <0.001 | <0.001 | <0.001 | <0.001 | 0.570 |
| LDLC, mmol/L | 2.15 ± 0.63 | 2.34 ± 0.93 | 3.54 ± 0.89 | 3.88 ± 0.56 | 2.40 ± 0.87 | 2.18 ± 0.81 | 0.005 | <0.001 | <0.001 | <0.001 | 0.660 |
| TG, mmol/L | 0.94 ± 0.37 | 2.49 ± 1.98 | 2.16 (2.06) | 1.98 ± 0.97 | 3.38 ± 2.10 | 2.48 ± 1.75 | <0.001 | <0.001 | <0.001 | <0.001 | <0.001 |
| HDLC, mmol/L | 1.35 ± 0.30 | 0.92 ± 0.38 | 1.17 ± 0.43 | 1.12 ± 0.32 | 0.89 ± 0.41 | 0.80 ± 0.15 | <0.001 | 0.003 | <0.001 | <0.001 | <0.001 |
| Hypertension^g^ | 79 (81) | 209 (170) | 35 (28) | 32 (25) | 130 (99) | 169 (140) | 0.220 | 0.406 | 0.380 | 0.150 | 0.274 |
| Diabetes^h^ | 41 (119) | 124 (255) | 21 (42) | 14 (43) | 85 (144) | 107 (202) | 0.103 | 0.247 | 0.874 | 0.017 | 0.047 |

^a^TC, high TC group. ^b^LDLC, high LDLC group. ^c^TG, high TG group. ^d^HDLC, low HDLC group. ^e^mean ± standard deviation. ^f^median (interquartile range). ^g, h^yes (no). BMI, body mass index. DBP, diastolic blood pressure. Diabetes, refers to whether combined with diabetes. FBS, fasting blood sugar. Hypertension, refers to whether combined with hypertension. SBP, systolic blood pressure. *P_D_-value,* the *P-values* for the dyslipidemia group. *P-value* < 0.05 indicated statistically significant difference.
